# Concordance between single-slice abdominal computed tomography-based and bioelectrical impedance-based analysis of body composition in a prospective study

**DOI:** 10.1007/s00330-025-11746-3

**Published:** 2025-06-19

**Authors:** Uli Fehrenbach, Clarissa Hosse, William Wienbrandt, Thula Walter-Rittel, Johannes Kolck, Timo Alexander Auer, Elisabeth Blüthner, Frank Tacke, Nick Lasse Beetz, Dominik Geisel

**Affiliations:** 1 Charité—Universitätsmedizin Berlin, Corporate Member of Freie Universität Berlin and Humboldt-Universität zu Berlin, Department of Radiology, Berlin, Germany; 2 Berlin Institute of Health at Charité—Universitätsmedizin Berlin, BIH Biomedical Innovation Academy, Berlin, Germany; 3 Charité—Universitätsmedizin Berlin, Corporate Member of Freie Universität Berlin and Humboldt-Universität zu Berlin, Department of Hepatology & Gastroenterology, Campus Virchow Klinikum and Campus Charité Mitte, Berlin, Germany

**Keywords:** Body composition, Computed tomography, Artificial intelligence, Bioelectrical impedance, Frailty

## Abstract

**Objectives:**

Body composition analysis (BCA) is a recognized indicator of patient frailty. Apart from the established bioelectrical impedance analysis (BIA), computed tomography (CT)-derived BCA is being increasingly explored. The aim of this prospective study was to directly compare BCA obtained from BIA and CT.

**Materials and methods:**

A total of 210 consecutive patients scheduled for CT, including a high proportion of cancer patients, were prospectively enrolled. Immediately prior to the CT scan, all patients underwent BIA. CT-based BCA was performed using a single-slice AI tool for automated detection and segmentation at the level of the third lumbar vertebra (L3). BIA-based parameters, body fat mass (BFM_BIA_) and skeletal muscle mass (SMM_BIA_), CT-based parameters, subcutaneous and visceral adipose tissue area (SATA_CT_ and VATA_CT_) and total abdominal muscle area (TAMA_CT_) were determined. Indices were calculated by normalizing the BIA and CT parameters to patient’s weight (body fat percentage (BFP_BIA_) and body fat index (BFI_CT_)) or height (skeletal muscle index (SMI_BIA_) and lumbar skeletal muscle index (LSMI_CT_)).

**Results:**

Parameters representing fat, BFM_BIA_ and SATA_CT_ + VATA_CT_, and parameters representing muscle tissue, SMM_BIA_ and TAMA_CT_, showed strong correlations in female (fat: *r* = 0.95; muscle: *r* = 0.72; *p* < 0.001) and male (fat: *r* = 0.91; muscle: *r* = 0.71; *p* < 0.001) patients. Linear regression analysis was statistically significant (fat: *R*^2^ = 0.73 (female) and 0.74 (male); muscle: *R*^2^ = 0.56 (female) and 0.56 (male); *p* < 0.001), showing that BFI_CT_ and LSMI_CT_ allowed prediction of BFP_BIA_ and SMI_BIA_ for both sexes.

**Conclusion:**

CT-based BCA strongly correlates with BIA results and yields quantitative results for BFP and SMI comparable to the existing gold standard.

**Key Points:**

***Question*** *CT-based body composition analysis (BCA) is moving more and more into clinical focus, but validation against established methods is lacking.*

***Findings*** *Fully automated CT-based BCA correlates very strongly with guideline-accepted bioelectrical impedance analysis (BIA).*

***Clinical relevance*** *BCA is currently moving further into clinical focus to improve assessment of patient frailty and individualize therapies accordingly. Comparability with established BIA strengthens the value of CT-based BCA and supports its translation into clinical routine.*

**Graphical Abstract:**

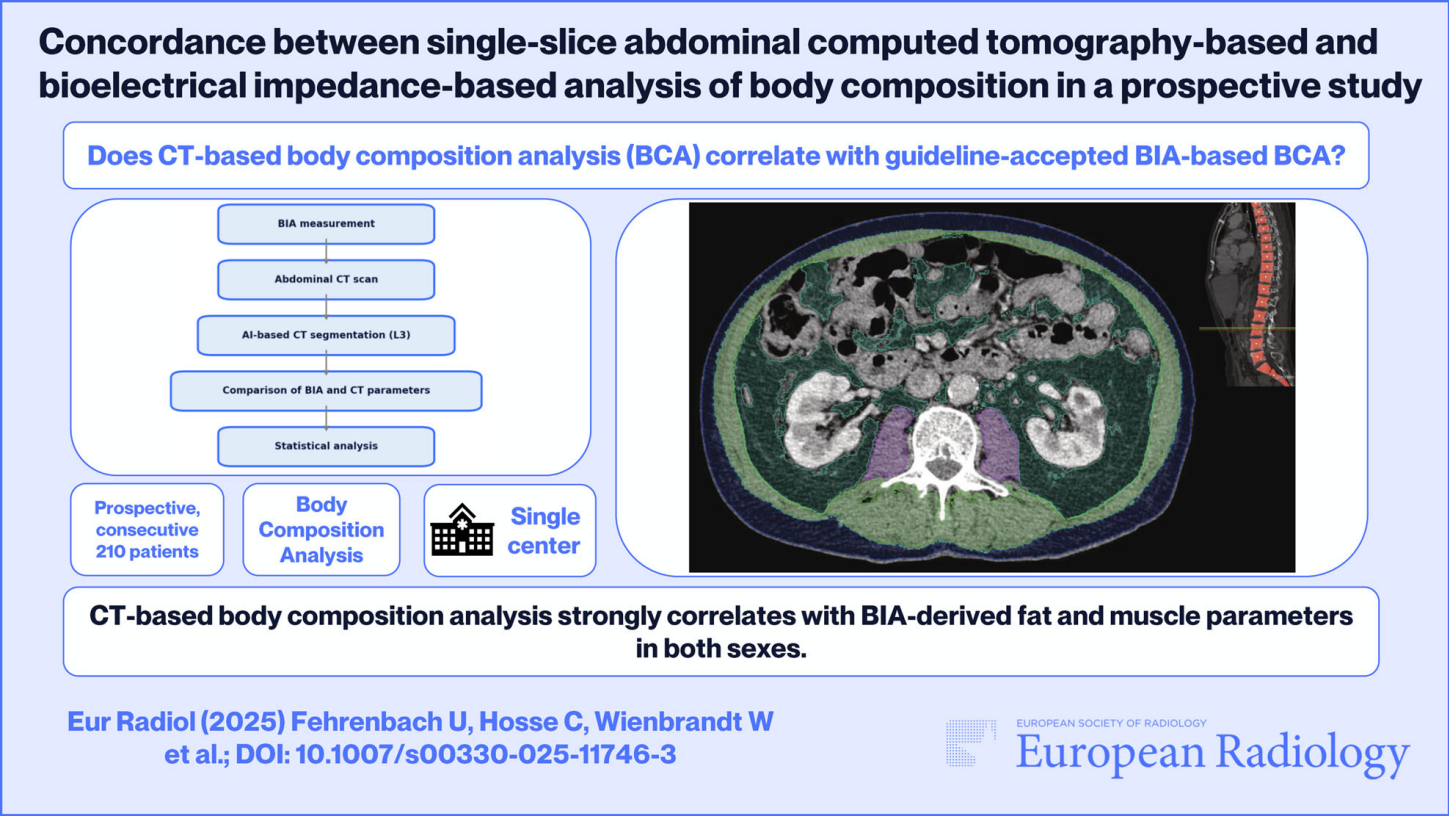

## Introduction

Although imaging studies facilitate opportunistic screening, their full potential has yet to be realized. One of the most relevant opportunistic uses of imaging information is body composition analysis (BCA). BCA measures the distribution of muscle, bone, and adipose tissue in the human body and is increasingly used to identify pathological conditions such as sarcopenia, cachexia, and myosteatosis [1–3]. While the body mass index (BMI) is widely used for initial risk assessment, it does not provide information on the relative proportions of different tissue types that contribute to a person’s overall weight. Therefore, two patients of the same sex and age may have the same weight and BMI, but their body composition and relative amounts of fat and muscle tissues may be entirely different [4, 5].

An alternative to BMI is bioelectrical impedance analysis (BIA), which distinguishes between fat-free body mass and the amount of body fat by subtracting the former from body weight. Imaging datasets obtained by techniques such as computed tomography (CT) and magnetic resonance imaging (MRI) can be used to quantify different tissue types through segmentation. Several methods for analyzing body composition using imaging have been proposed. Initially, segmentation of the psoas muscle was used as an indicator, but due to the limitations of the simplified measurement of a single muscle, more sophisticated segmentations have been established in the meantime, and single-slice segmentation of adipose and muscle tissue at the level of L3 has become the most commonly used imaging-based BCA method [6–8]. This method differentiates the relative proportions of muscle and visceral and subcutaneous fat and has given rise to the lumbar skeletal muscle index (LSMI), which normalizes muscle-based measurement with body height and can be used to detect sarcopenia [9].

BCA can identify frail patients, such as those with sarcopenia or sarcopenic obesity, who may have a normal BMI but present with reduced muscle mass and obesity [10]. The clinical relevance of BCA has been demonstrated in multiple studies and clinical scenarios, and various segmentation methods for imaging-based BCA, some of them using artificial intelligence (AI), have been developed to automate otherwise time-consuming manual segmentation [11–15]. However, most of these studies were retrospective, and there is a need to validate CT-based BCA with established standards [16]. This prospective study hypothesizes that AI-based image segmentation from CT is equivalent to established BIA for assessing body composition in a non-selected cohort of adult individuals. We prospectively analyzed and compared BCA using both AI-based CT image segmentation and BIA in patients undergoing routine clinical CT imaging.

## Materials and methods

### Study design

**Fig. 1:**
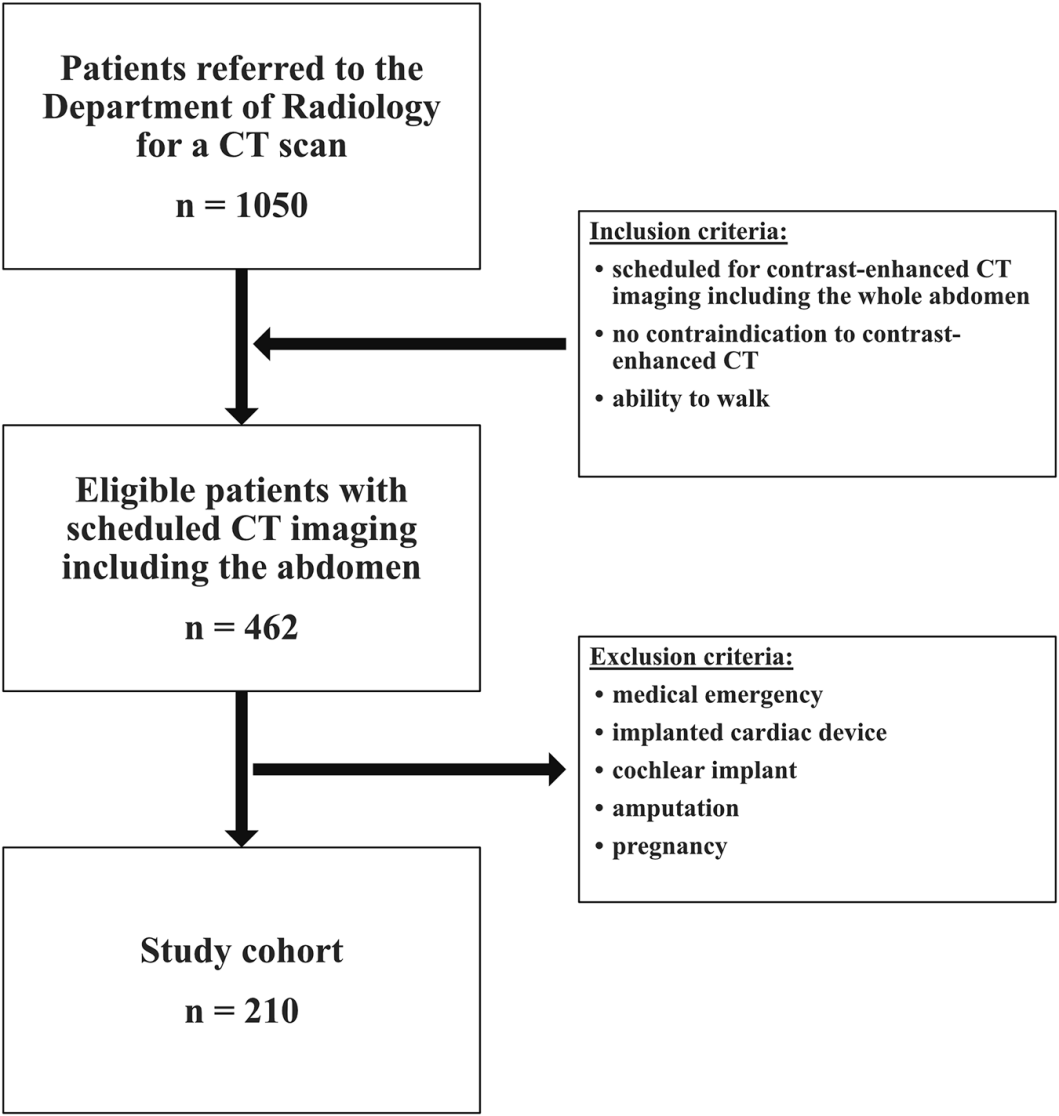
Flowchart depicting the inclusion and exclusion criteria for this study

In this prospective single-centre study we analyzed body composition using AI-based CT image segmentation and BIA. Consecutive patients that were referred to a non-emergency abdominal CT scan were included into the study, according to predefined criteria (Fig. 1). This study was approved by the institutional review board (reference number: EA1/094/22) and conducted in accordance with the declaration of Helsinki.

### Patient recruitment and image acquisition

We enrolled adult patients (18 years and older) who underwent a scheduled contrast-enhanced CT scan, including the whole abdomen, and were able to stand unassisted. Patients were enrolled between May 2021 and July 2021 without any disease-based preselection, excluding those with time-critical medical emergencies, implanted cardiac devices, cochlear implants, pregnancy, and amputation of one or more extremities, as these patients cannot or should not undergo BIA. Patients unable to stand upright without help while holding BIA controllers in both hands for approximately 60 s were also excluded. Before CT imaging, patients were informed about the purpose, design, temporal sequence, and potential benefits of the study and offered the opportunity to participate. The enrollment rate for this prospective study was 84%, and written informed consent was obtained from all patients before BCA. A flowchart depicting the inclusion process for this study is shown in Fig. 1.

For the study examinations we used the GE Revolution HD (GE Healthcare). Every protocol consisted of 100 kVp voltage, 20–120 mA tube current, recon section interval of 1.25 to 5 mm. The protocol is a standardized protocol that can be used on almost all devices. We used examinations with and without contrast agent.

### Body composition analysis using bioelectrical impedance analysis

BIA-based BCA involved instructing participants not to drink or eat for at least two hours before the measurement started and to remove any metal accessories such as jewelry, watches, and electrical devices. They were asked to stand barefoot and upright on the BIA´s ground electrodes and to adjust their heels around the sensor pedals. During the test, patients had to hold the BIA controller-connected electrodes with both hands. Patients needed to be silent for the total period of measurement. Body height was measured with a calibrated stadiometer. All measurements were taken sequentially and in the same order (height, BIA, CT examination) within 1.5 h of a single visit. The InBody 770 (InBody Europe B.V.) was used to take all BIA measurements using the manufacturer’s recommended technique, and appropriate BIA equations were used to calculate the parameters body fat mass (BFM_BIA_), skeletal muscle mass (SMM_BIA_), and visceral fat area (VFA_BIA_). The SMI_BIA_ was calculated using the following formula: SMM_BIA_ (kg)/height^2^ (m^2^). Body fat percentage (BFP_BIA_) was calculated using the formula: weight (kg)/BFM_BIA_ * 100.

### Body composition analysis using fully automated CT image segmentation

**Fig. 2:**
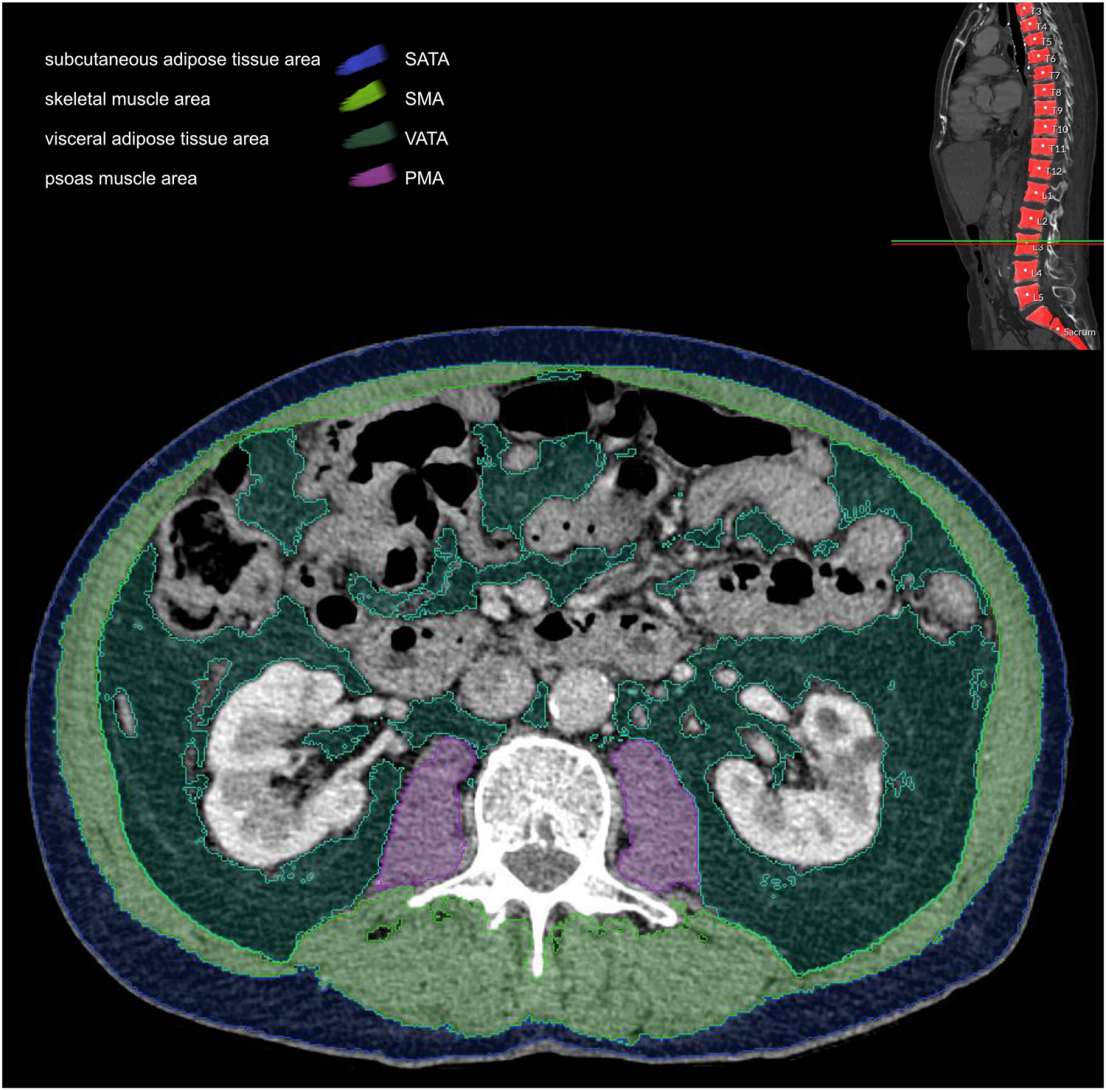
Example illustration of the automated CT-based body composition analysis. At the top right, the automated localization of L3 is included

For CT-based BCA, an AI-based image segmentation tool that had previously been internally and externally validated and is fully integrated in our PACS (Visage version 7.1., Visage Imaging GmbH) was used. The tool was first described by Beetz et al [14]. It is based on a convolutional neural network, U-net, and consists of nine blocks. Initial training data consisted of 200 axial CT images of the L3 level, and augmentation was applied during training to improve generalization of the network. The areas (in cm^2^) of the different tissue classes of interest included visceral adipose tissue (VATA_CT_), subcutaneous adipose tissue (SATA_CT_), psoas muscle (PMA_CT_), and skeletal muscle (SMA_CT_) and were automatically calculated and coded with different colors. All AI-based image segmentations performed on study patients were checked by a radiologist with over five years of experience in abdominal CT imaging. No manual correction was needed. Total abdominal muscle area (TAMA_CT_) was calculated by adding PMA_CT_ + SMA_CT_. The LSMI_CT_ was calculated using the established formula: TAMA_CT_ (cm^2^)/height^2^ (m^2^) [9]. Body fat index_CT_ (BFI_CT_) was calculated by normalizing the adipose tissue area by the patient’s weight (for comparability with BFP_BIA_) using the following formula: VATA_CT_ + SATA_CT_ (cm^2^)/weight (kg). An example of automated segmentation in CT-based BCA is illustrated in Fig. 2.

### Statistical analysis

Statistical and data analyses were performed using Stata/MP 17.0 (StataCorp). Data were tested for normal distribution and parametric test procedures were consecutively applied. Descriptive statistics for numerical variables were expressed as mean and standard deviation. T-tests were used to compare differences in means between groups. Pearson correlation coefficients were used for correlation analysis between two continuous variables. Additionally, linear regression was utilized to determine if CT-based indices could predict BIA-based indices. The results were presented as scatter plots. Furthermore, agreement between BFP was assessed using Bland-Altman plots. BFP from CT was calculated using a linear regression model with visceral and subcutaneous fat area, height, weight, and gender. Results with a *p*-value < 0.05 were considered statistically significant.

## Results

### Baseline data

A total of 210 patients with a mean age of 60 ± 14 years (ranging from 19 to 91 years) were prospectively enrolled. The study population consisted of slightly more male (55%) than female (45%) patients. Mean weight was 75 ± 17 kg, and mean height was 171 ± 9 cm, resulting in a mean BMI of 26 ± 5 kg/m^2^. The main indication for CT was cancer baseline or follow-up imaging in 185 cases (88%). Clinical characteristics are compiled in Table 1.

**Table 1:** Patient characteristics

	Total (*n* = 210)
Age, years^a^	60 ± 14
Sex, *n* (%)	
Female	94 (45%)
Male	116 (55%)
BMI^a^	26 ± 5
Indication for CT, *n* (%)	
Cancer	185 (88%)
Infection	4 (1.9%)
Cardiovascular disease	4 (1.9%)
Acute abdominal pain	4 (1.9%)
Other	13 (6%)

*BMI* body mass index, *CT* computed tomography

^a^ Mean ± standard deviation

### Body composition analysis

No manual adjustment was required by the radiologist checking the fully automated CT image segmentation of the AI-algorithm. Quantitative results for BIA-based and CT-based BCA parameters are compiled in Tables 2 and 3.

**Table 2:** Results of BIA-based body composition analysis

	All (*n* = 210)	Female (*n* = 94)	Male (*n* = 116)	*p*-value
BFM_BIA_ (kg)	22.3 ± 11.0	24.1 ± 11.6	20.8 ± 10.3	*p* = 0.031
SMM_BIA_ (kg)	28.6 ± 6.2	23.4 ± 3.7	32.8 ± 4.5	*p* < 0.001
VFA_BIA_ (cm^2^)	110.6 ± 53.7	121.5 ± 56.0	100.8 ± 49.9	*p* = 0.003
BFP_BIA_ (%)	28.8 ± 10.1	33.9 ± 9.3	24.7 ± 98.7	*p* < 0.001
SMI_BIA_ (kg/m^2^)	9.7 ± 1.5	8.7 ± 1.2	10.5 ± 1.1	*p* < 0.001

*BFM* body fat mass, *SMM* skeletal muscle mass, *VFA* visceral fat area, *BFP* body fat percentage, *SMI* skeletal muscle index

**Table 3:** Results of CT-based body composition analysis

	All (*n* = 210)	Female (*n* = 94)	Male (*n* = 116)	*p*-value
VATA_CT_ (cm^2^)	109.2 ± 99.5	68.1 ± 59.6	142.4 ± 112.4	*p* < 0.001
SATA_CT_ (cm^2^)	176.8 ± 104.1	200.5 ± 116.9	157.5 ± 88.3	*p* = 0.003
PMA_CT_ (cm^2^)	16.5 ± 6.0	12.0 ± 3.3	20.1 ± 5.1	*p* < 0.001
TAMA_CT_ (cm^2^)	136.4 ± 34.0	110.3 ± 20.1	157.6 ± 27.6	*p* < 0.001
BFI_CT_ (cm^2^/kg)	3.6 ± 1.6	3.7 ± 1.5	3.5 ± 1.7	*p* = 0.371
LSMI_CT_ (cm^2^/m^2^)	46.4 ± 9.9	41.1 ± 8.1	50.7 ± 9.1	*p* < 0.001

*VATA* visceral adipose tissue area, *SATA* subcutaneous adipose tissue area, *PMA* psoas muscle area, *TAMA* total abdominal muscle area, *BFI* body fat index, *LSMI* lumbar skeletal muscle index

### Comparison of BIA-based and CT-based BCA parameters/indices

**Fig. 3:**
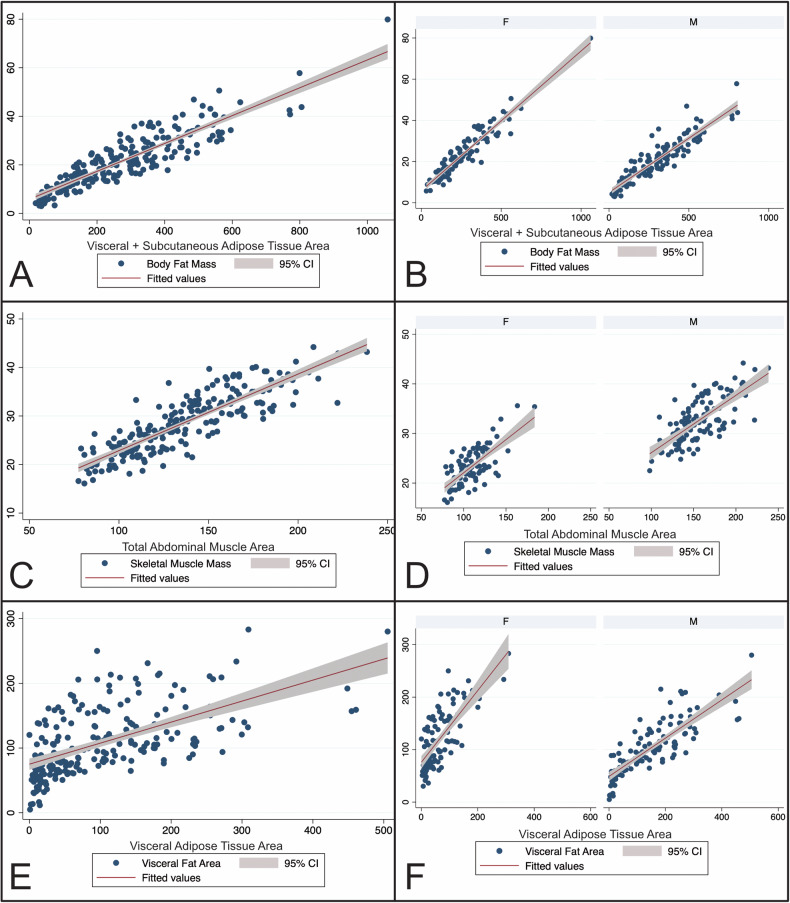
Scatter plots of the correlation analysis between BIA-based and CT-based BCA parameters. Visceral and subcutaneous adipose tissue area and body fat mass: **A** total study population, **B** gender-specific subgroup analysis; total abdominal muscle area and skeletal muscle mass: **C** total population, **D** gender-specific subgroup analysis; visceral adipose tissue area and visceral fat area: **E** overall cohort, **F** gender-specific subgroup analysis

There were highly significant positive correlations between BIA- and CT-based BCA parameters of adipose and muscle tissue. BFM_BIA_ and VATA_CT_ (all: *r* = 0.63; female: *r* = 0.74; male: *r* = 0.82), SATA_CT_ (all: *r* = 0.87; female *r* = 0.93; male *r* = 0.80) as well as VATA_CT_ + SATA_CT_ combined (all: *r* = 0.89; female *r* = 0.95; male *r* = 0.91) showed very strong and strong significant correlations in the total study population and in the gender-specific subgroup analysis (*p* < 0.001) (Fig. 3A, B). SMM_BIA_ and PMA_CT_ (*r* = 0.73) and TAMA_CT_ (*r* = 0.86) showed strong, significant correlations in the total population (*p* < 0.001). Gender-specific correlation of SMM_BIA_ and PMA_CT_ showed a weak, significant correlation in females (*r* = 0.38) and a moderate correlation in males (*r* = 0.51) (both *p* < 0.001). Strong significant correlations between SMM_BIA_ and TAMA_CT_ were found in both females (*r* = 0.72) and males (*r* = 0.71) (both *p* < 0.001) (Fig. 3C, D). VFA_BIA_ and VAT_CT_ (*r* = 0.60, *p* < 0.001) showed moderately significant correlations in the total population and strong correlations in the gender-specific subgroup analysis (female *r* = 0.73; male *r* = 0.82; *p* < 0.001) (Fig. 3E, F).

**Fig. 4:**
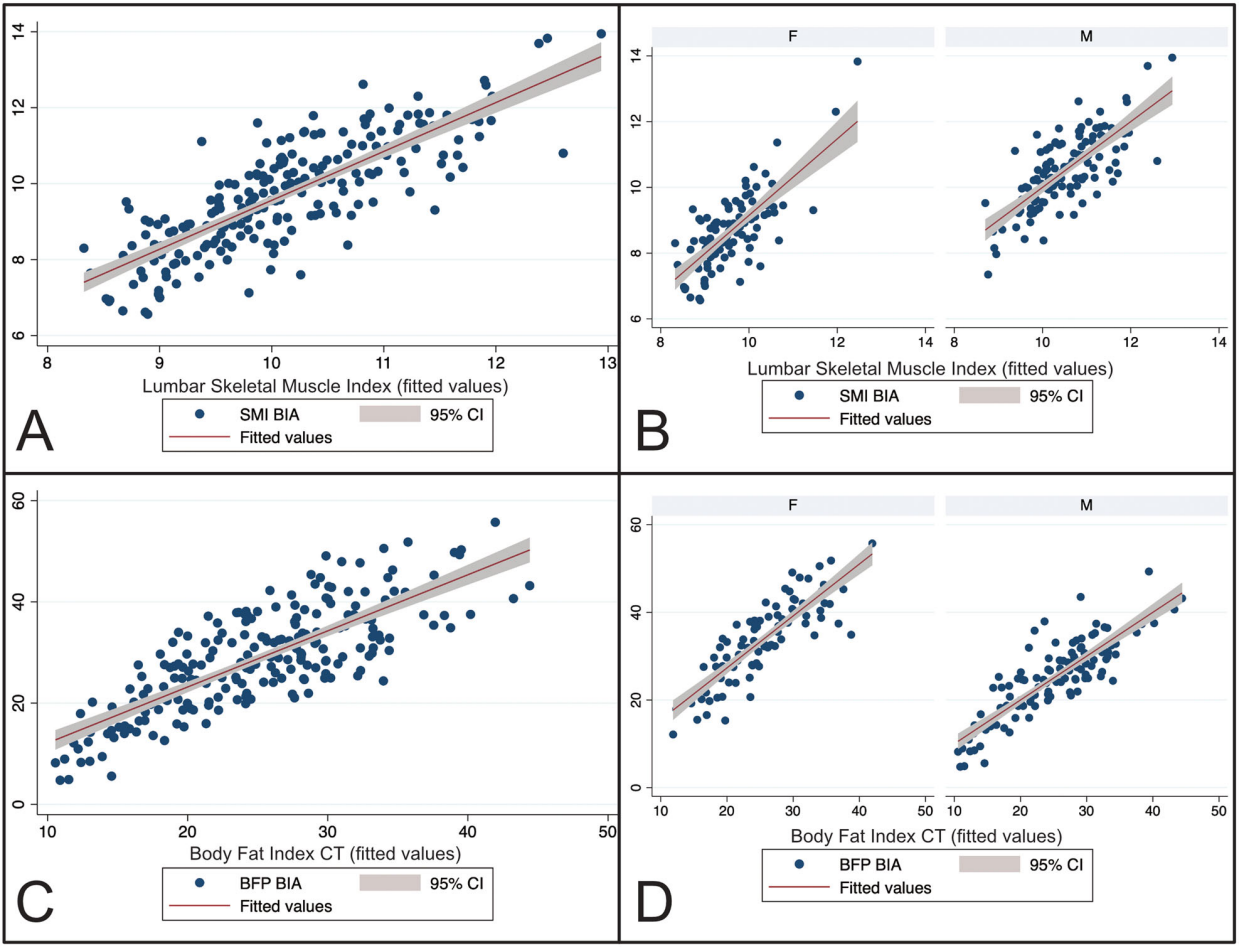
Scatter plots of the correlation analysis between BIA-based and CT-based BCA indices. Lumbar skeletal muscle index and skeletal muscle index: **A** total population, **B** gender-specific subgroup analysis; body fat index and body fat percentage: **C** total population, **D** gender-specific subgroup analysis

**Fig. 5:**
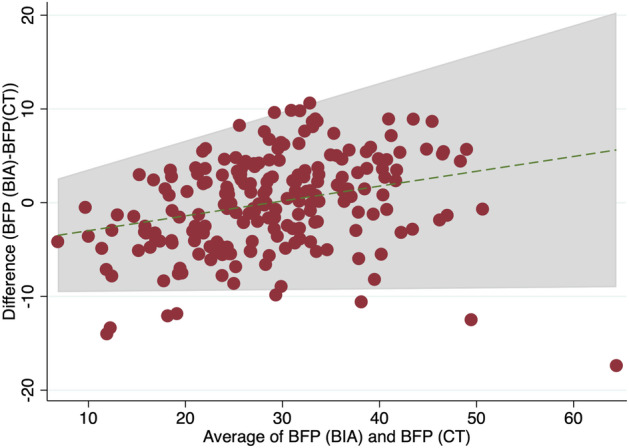
Bland–Altman plot comparing body fat percentage determined from BIA with body fat percentage predicted from CT (prediction from linear progression using subcutaneous and visceral fat area, height, weight, and gender). 4.76% or 10/210 are outside of the limits of agreement; mean difference −4.57 + 0.16*average

Indices SMI_BIA_ and LSMI_CT_ (*r* = 0.81) as well as BFP_BIA_ and BFI_CT_ (*r* = 0.79) showed strong significant correlations (*p* < 0.001). Linear regression was used to test whether CT-based indices could predict BIA-based indices. Overall regression of SMI_BIA_ and LSMI_CT_ (*R*^2^ = 0.66) as well as of BFP_BIA_ and BFI_CT_ (*R*^2^ = 0.62) was statistically significant (both *p* < 0.001). In gender-specific subgroup analysis, linear regression analysis remained significant for both sexes (*p* < 0.001, Table 4 and Fig. 4). Bland-Altman plots showed excellent agreement for BFP derived from BIA with BFP predicted from CT with 4.76% or 10/210 patients outside of the limits of agreement (Fig. 5).

**Table 4:** Linear regression analysis of BIA-based indices and CT-based indices representing adipose and muscle tissue

	Predictor	Response	*R*^2^-value	*β*-value	*p*-value
All (*n* = 210)	LSMI	SMI	0.66	0.81	< 0.001
Female (*n* = 94)	LSMI	SMI	0.56	0.75	< 0.001
Male (*n* = 116)	LSMI	SMI	0.56	0.75	< 0.001
All (*n* = 210)	BFI_CT_	BFP	0.62	0.79	< 0.001
Female (*n* = 94)	BFI_CT_	BFP	0.73	0.86	< 0.001
Male (*n* = 116)	BFI_CT_	BFP	0.74	0.86	< 0.001

*LSMI* lumbar skeletal muscle index, *SMI* skeletal muscle index, *BFI*_CT_ body fat index, *BFP* body fat percentage

**Fig. 6:**
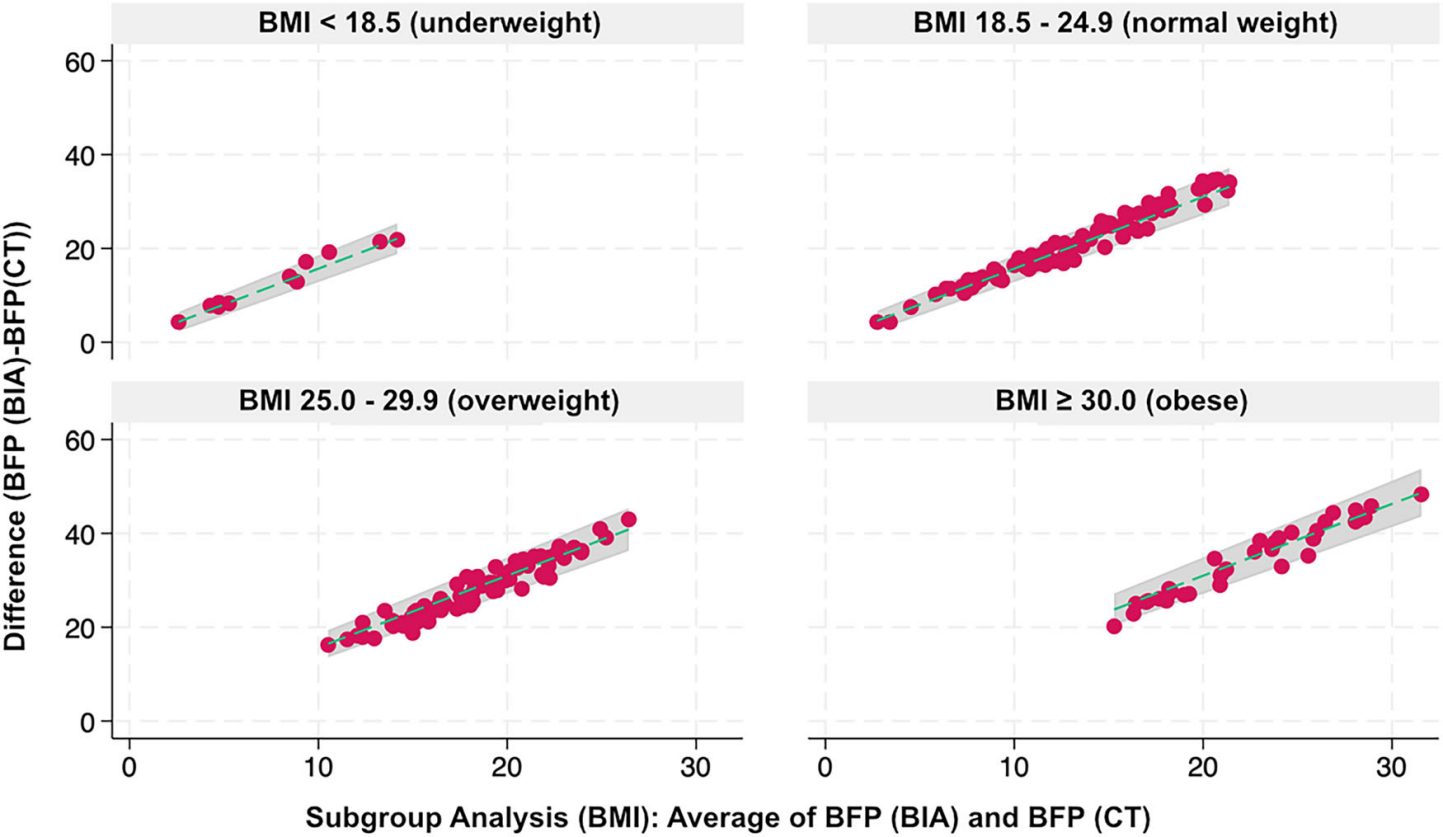
Bland-Altman plots displaying the difference in body fat percentage measurements between bioelectrical impedance analysis (BIA) and computed tomography (CT) across BMI categories (< 18.5, 18.5–24.9, 25.0–29.9, ≥ 30). The *x*-axis represents the average of the two methods, while the *y*-axis shows their difference

Analyzing subgroups based on different BMI categories offers insights into variations in body composition analysis across different weight ranges (Fig. 6).

## Discussion

In this prospective study, we directly compared BCA using BIA and fully automated CT-based single-slice segmentation by an AI tool in a non-selected patient cohort that included patients from a large university hospital in Germany. We found highly significant correlations between the two BCA methods for both adipose and muscle tissue.

While BIA is recognized as an accurate method for analyzing body composition, it can only indirectly calculate an individual´s muscle and fat mass [17]. BIA measurements can be impaired by several factors, including clothes, food and drink consumption, physical activity, medical conditions impacting fluid and electrolyte balance, cold temperature, and individual characteristics such as abdominal obesity, menstrual cycle, and menopause. Appropriate age-, population-, and pathology-specific BIA equations are also needed to determine SMI and BFP [18, 19]. Ethnicity may also have an impact on BIA measurement, highlighting the need for validation studies in diverse populations [20]. Moreover, BIA measurement requires additional effort with personnel expenses and is not feasible in every patient, e.g., patients with implanted cardiac devices as excluded from this study.

CT-based BCA could offer several clinical and practical benefits compared to BIA. First, the segmentation of individual tissue compartments based on imaging is generally unaffected by the aforementioned factors that compromise BIA. Additionally, by incorporating CT-based BCA of clinically indicated imaging into routine clinical practice, the need for additional resources required for BIA is effectively obviated. At the same time, the additive time required for segmentation is eliminated if fully automated BCA software can be used, so that repeated measurements during the course of the disease are also realistic [14, 21, 22]. Apart from measuring muscle areas, CT segmentation includes the assessment of tissue density, enabling the estimation of muscle quality and thus the recognition of pathological conditions like myosteatosis [23–27]. The clinical utility of CT-based BCA has been highlighted in numerous recent studies [12, 16, 28, 29]. Despite promising retrospective analyses, there exists a lack of validation for CT-based BCA when compared to other more established BCA. It should be noted that both this study and its conclusions are based on a radiology-ready healthcare setting where CT imaging is readily available. In contrast, for healthcare centers without access to CT imaging, BIA remains a guideline-accepted and widely used method for BCA.

In imaging, opportunistic screening refers to the detection of unexpected findings unrelated to the indications for which an imaging test, such as CT, is performed [2]. Similar to an additional blood pressure measurement when visiting a general practitioner’s office for other reasons, imaging can be used to look for additional relevant findings, such as pulmonary nodules, aortic aneurysms, and bony changes in individuals undergoing chest CT to evaluate the lung parenchyma [30]. Opportunistic screening in CT imaging makes use of previously largely unused imaging data contained in chest and abdominal CT scans, which are generally unrelated to the specific clinical indication. This occult information from imaging can thus be used to improve disease prevention, help create risk profiles, and potentially aid in the diagnosis of relevant pre-symptomatic diseases [1]. CT-based BCA can also be useful in clinical routine. Critically ill patients in the ICU, who are especially susceptible to rapid and significant muscle mass loss, would likely benefit from the application of CT-based BCA, though various conditions of ICU patients, as edema or fluid overload, affect the accuracy of BIA. CT-based BCA offers the potential of a more accurate monitoring of muscle wasting over time [31]. CT-based BCA could be employed to monitor the effectiveness of physical therapy interventions in the ICU. By comparing body composition data obtained before and after therapy, clinicians could assess the extent of muscle recovery or continued loss, providing critical information for adjusting therapy regimens. This capability would be particularly useful for detecting subtle changes in muscle mass that might not be apparent through clinical observation alone. Repeated assessments using CT-based BCA could guide dynamic adjustments to therapy protocols, ensuring that interventions remain effective as the patient’s condition evolves. As such, the clinical utility of BIA-based BCA has already been studied prospectively in several diseases [32–34]. A few smaller studies prospectively comparing BIA and CT-based BCA have been published, showing correlations between the two methods [35–38]. Our study differs from these studies, especially the study design, since we directly compared BIA and CT examinations. Other unique features of our study are the size and diversity of the study population. In addition, our study adds to the existing body of data on automated CT-based BCA, which we believe can enable its translation into routine clinical practice.

Gender-specific subgroup analysis revealed consistently strong correlations between BIA- and CT-based body composition parameters across both sexes, although the strength of association varied between male and female patients. Differences were more pronounced in the subgroup analyses when only specific compartments, such as visceral adipose tissue or the psoas muscle were considered. This phenomenon likely reflects the overall sex-specific distribution patterns of adipose and muscle tissue. However, when total muscle or fat area was included in the analysis, the strength of associations remained largely comparable between sexes. These findings highlight the importance of sex-specific evaluation in body composition analysis, as physiological differences between male and female patients may influence the accuracy and interpretability of imaging- and BIA-derived indices.

And yet, CT-based BCA also has some limitations. Even though the average radiation dose has substantially decreased with state-of-the-art CT scanners and optimized CT protocols, CT imaging can only be performed if it is clinically indicated, which precludes its widespread use [39, 40]. Compared to semiautomatic BCA tools, fully automated segmentation is much faster and can therefore be used in larger study populations, but its results still require verification by an experienced radiologist and may need to be manually corrected in individual cases. Moreover, the accuracy of automated image segmentation might also be limited in patients with foreign material, severe ascites, edema, or mesenteric fat stranding, as the altered radiodensity could degrade the capacity of the tool to correctly differentiate adipose tissue from muscle tissue [41, 42]. However, our study highlights the robustness of automated segmentation, as no manual adjustments had to be made in any of our consecutively selected patients. BCA was conducted using an AI-driven single-slice tool for automated detection and segmentation at the level of L3. Recent research shows that single-slice L3 cross-sectional areas and multi-slice T12–L5 abdominal volumes for skeletal muscle, visceral adipose tissue and subcutaneous adipose tissue show strong correlations. The associations between these areas and volume measurements with all-cause mortality are comparable, indicating that both methods are equally effective for use in population studies when body composition is assessed at a single timepoint [43]. Accordingly, using the single-slice method is the simpler and faster option with comparable results. Additionally, BIA was selected as the reference standard due to its widespread use in nutritional science, internal medicine, and sports medicine.

Despite its strengths, this study has several limitations that should be acknowledged. This study has a single-center design. Conducting the study within a single institution limits the generalizability of the findings, as the patient population may not fully represent the broader demographic and clinical variability seen in other regions or healthcare settings. The specific characteristics of the patient cohort, such as ethnic background, socioeconomic status, and prevalent health conditions, may influence body composition parameters and their correlation between BIA and CT-based BCA. In our university clinic, a high proportion of the patients examined are cancer patients, which is reflected in the study population. Only emergency examinations and assessments of critically ill patients who were unable to undergo the additional BIA were excluded. Additionally, the use of a single AI tool for CT-based BCA may introduce bias specific to the software’s algorithm, which might differ in performance when applied in other clinical environments or when using different tools. Therefore, the results should be interpreted with caution when extrapolating to diverse populations or settings, and further multicenter studies are warranted to validate these findings across different patient populations and clinical practices.

In conclusion, our study demonstrates strong correlations between BIA-based and CT-based BCA in a prospective study design. Thus, an additional BIA appears unnecessary for patients who have undergone an abdominal CT scan as part of their clinical workup. Our study underscores the validity of CT-based BCA and suggests routine dissemination of this valuable information, which would otherwise go unused.

## Abbreviations

AI: Artificial intelligence
BFI: Body fat index
BFM: Body fat mass
BFP: Body fat percentage
BMI: Body mass index
CT: Computed tomography
L3: Third lumbar vertebral body
LSMI: Lumbar skeletal muscle index
SATA: Subcutaneous adipose tissue area
SMI: Skeletal muscle index
SMM: Skeletal muscle mass
VATA: Visceral adipose tissue area
